# Alveolar Echinococcosis of the Liver: Report of Three Cases From Different Geographic Areas of Iran

**DOI:** 10.5812/hepatmon.6143

**Published:** 2012-09-05

**Authors:** Bita Geramizadeh, Saman Nikeghbalian, Seyed Ali Malekhosseini

**Affiliations:** 1 Transplant Research Center, Department of Pathology, Shiraz University of Medical Science, Shiraz, IR Iran; 2 Department of Pathology, Department of Pathology, Shiraz University of Medical Science, Shiraz, IR Iran; 3 Transplant Ward, Department of Surgery, Shiraz University of Medical Science, Shiraz, IR Iran

**Keywords:** Alveolar Echinoccosis, Echinococcus Multilocularis, liver, Iran

## Abstract

**Introduction:**

Alveolar echinococcosis (AE) is a chronic, serious and sometimes lethal parasitic infection, which is caused by Echinococcus multilocularis (EM). AE has been reported to occur in people from the north of Iran; however, until now there have been no cases of AE reportedfrom the southern provinces, such as Khuzestan.

**Case Presentation:**

Herein, we report our experiences with three cases of hepatic AE, who presented with large masses in the liver, from both the northern and southern provinces of Iran. Three patients are described who were presented with hepatic masses from different provinces of the country.

**Conclusions:**

There were three female patients, 21, 47 and 53 year-old. They were presented with liver masses from different centers of the country i.e. Khorasan, Ardabil and Khuzestan. According to our experience, AE is not an uncommon disease in Iran. Moreover, it has a widespread epidemiology, i.e., this disease should be suspected in all provinces of Iran, not only in the northern, but also in the southern regions of the country.

## Introduction

Hepatic alveolar hydatid cysts caused by Echinococcus multilocularis (EM), is a very rare and severe disease, caused by the growth of its larval stage in the liver (1). Epidemiologically, the most common geographic areas of this parasitic infestation are in the northern hemisphere, occurring in parts of Europe, Asia, Alaska, and North America (2). There have previously been a few reports of this infestation from Iran (3). These reports show the presence of EM in the northern parts of the country, including the northwestern provinces of Azerbaijan (4) and Ardebil (5) as well as the northeastern province of Khorasan (6).

## Case Presentation

Until now, no case of AE of the liver has been reported from the south of Iran. In this study, we report our experience with three cases of hepatic AE from Ardebil, Khorasan and Khuzestan provinces.

### 2.1. CASE No. 1

A 21-year-old woman from the Ardebil Province presented with complaints of jaundice and abdominal distension, which had been occurring during the past two years, she was referred to our center due to an inoperable, large liver mass. Her past medical history was unremarkable. She had grown up on a farm in a rural area.

### 2.2. Case No. 2

A 53-year-old female patient from the Khorasan Province was referred to the hepatobiliary surgeon. She had experienced; right upper quadrant (RUQ) pain, fever and dyspepsia for the last four months.

### 2.3. Case No. 3

A 47-year-old lady from Khuzestan, who had grown up on a farm was referred to us with; fever, jaundice and abdominal pain. 

## Discussion

### 3.1. Case No. 1

Physical examination showed icteric sclera. Heart and lung examination were unremarkable. Abdominal examination showed a mild tenderness in the RUQ, with mild hepatomegaly. Vital signs were normal. The patient was not febrile. Laboratory examination showed; leukocytosis, anemia, hyperbilirubinemia and high ALT (alanine aminotransferase), AST (aspartate aminotransferase) and alkaline phosphatase levels (Table 1 shows a summary of clinical and paraclinical characteristics). Abdominal computerized tomography (CT)and magnetic resonance imaging (MRI), showed a large mass in the liver (images have been reported as a photo clinic) (7). A liver needle biopsy was performed, but this was reported to be unsatisfactory, with extensive necrosis. Subsequently a laparotomy was carried out, and this revealed a large mass in the hilar region, with adherence to the surrounding tissue. The mass was unremarkable, so a wedge biopsy was taken and the diagnosis of hepatic alveolar hydatid cyst was made. Albendazole medication was started right after the tissue diagnosis (10-12 mg/kg/day). It has now been approximately one year after therapy, she is well and symptom free.

**Table 1:** Summary of Clinical and Laboratory Findings in the Three Cases[Table-fn fn305]

	**Age, y**	**Clinical Presentation**	**Bilirubin a, mg/dl**	**ALT, IU/L**	**AST, IU/L**	**AIK, IU/L**	**WBC, ml**	**Hb, gr/dl**
**No. 1**	21	Juandice	4	60	78	220	135000	9
**No. 2**	53	Juandice fever	3.5	268	262	176	155000	10
**No. 3**	47	Juandice fever	4.5	102	75	744	4000	6.5

Abbreviations: ALT, alanine aminotransferase < 40IU/L; AST, aspartate aminotransferase < 40 IU/L; ALK, alkaline phosphatase < 150IU/L; Hb, hemoglobin: 12-16 gr/dl; WBC, white blood cell count: 4000-8000/ml.

^a^Bilirubin (normal < 1.5 gr/dl).

### 3.2. Case No. 2

**Figure 1:**
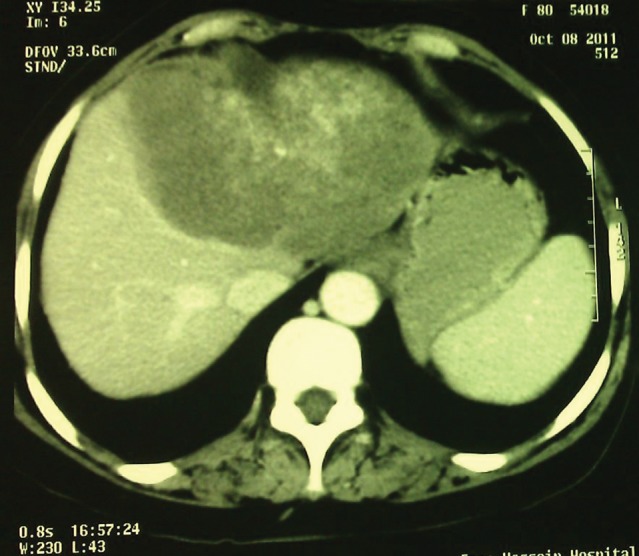
CT Scan Shows Multiloculated Mass in the Left Lobe of the Liver

**Figure 2:**
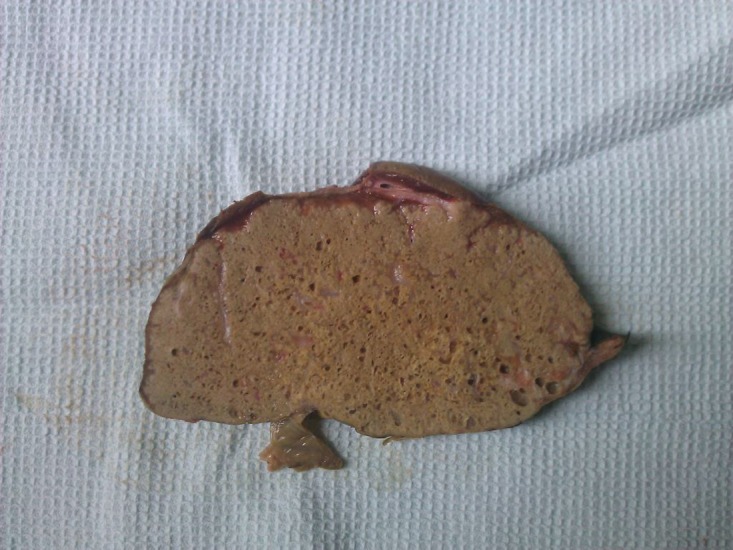
Gross View of the Resected Liver Mass With Tiny Sponge Like Cysts

**Figure 3:**
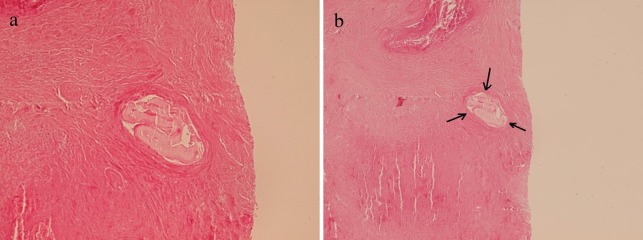
a and b Microscopic Sections From the Liver Mass Show Necrosis With Small Laminated Membranes (H&E x250).

Physical examination showed icteric sclera. Heart and lung examination were unremarkable. Abdominal examination showed mild hepatomegaly. Vital signs were normal. Laboratory findings showed; leukocytosis, hyperbilirubinemia, anemia and high AST, ALT and alkaline phosphatase levels (Table 1).Abdominal CT scan (Figure 1) showed a multiloculated mass in the left lobe of the liver. Liver needle biopsy in the local hospital was reported as the hydatid cyst of Echinococcus granulosus, so with this diagnosis, the patient was operated on and a segment of the liver was resected, this showed a large well-defined mass (20x16x7 cm) with tiny honeycomb like microcystic spaces (Figure 2). Pathologic studies showed complete necrosis, with some larval membranes (Figure 3).With the pathologic diagnosis of alveolar hydatid cyst, albendazole therapy was started. Now, approximately six months after surgery, the patient is doing well and is completely free of metastasis and symptoms.

### 3.3. Case No. 3

**Figure 4:**
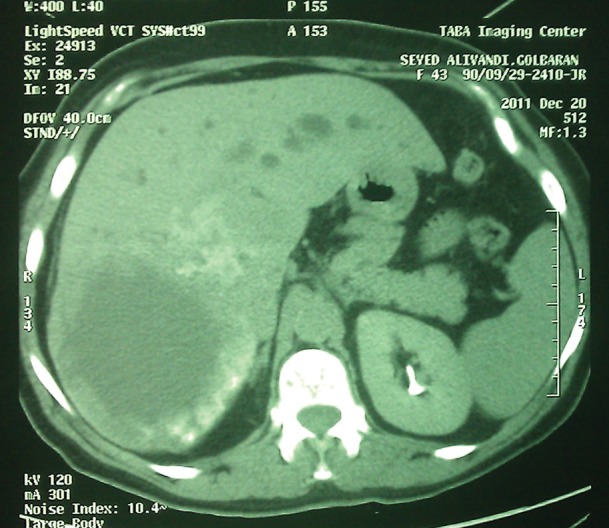
CT Scan Shows Well Defined Masses in Right Lobe of the Liver

Physical examination showed icteric sclera. Heart and lung examination were unremarkable. Abdominal examination showed hepatomegaly. Laboratory findings showed; anemia, hyperbilirubinemia and high AST and ALT levels (Table 1). Abdominal CT scan showed multiple, different sized and shaped masses, mostly in the right lobe, with the largest one measuring 10x10 cm (Figure 4).With possible diagnosis of an infiltrative lesion or metastasis, a liver biopsy was performed in the local hospital; unfortunately, this was reported to be insufficient because of the presence of extensive necrosis. Repeated biopsy in our center showed extensive necrosis and larval membranes. Review of the previous biopsy showed similar membranes, which had been missed. The patient was put on albendazole therapy before a decision concerning surgery could be made. Now after approximately two months, her abdominal pain has improved, and she has mild icteric sclera, to be followed-up by imaging studies for probable future surgery or liver transplant.

Hepatic alveolar echinococcosis is a rare, but potentially life-threatening parasitic disease, this develops as a result of intrahepatic growth of the Echinococcus multilocularis larvae (7, 8). Humansare accidental intermediate hosts, as rodents are the most common intermediate host, and carnivores are the final hosts. Carnivores such as foxes, can transmit the parasite to humans via their feces and the larvae develop in the liver, like a slow growing cancer (8). The definitive (final) hosts are commonly found in the northern hemisphere, occurring in parts of Europe, Asia, Alaska and North America (2). However, the majority of human cases of alveolar echinococcosis, have been reported in the People’s Republic of China (9). In Iran, all of the previously reported cases have been in the northeast and northwest provinces, i.e., Khorasan and Ardebil, respectively (3-6). During the last 15 years in our center, which is the referral center of hepatobiliary surgery and liver transplantation for the whole country, we have had three cases of hepatic alveolar hydatid cysts from the north and south of Iran. All of the patients were female, and all had come from rural areas, with a history of close contact with animals. One of our cases has been living in a rural area of the southern province of Khuzestan. Until now, no case of Echinococcus multilocularis has been reported from the south of Iran. The most common presenting symptoms of liver involvement in alveolar hydatid cyst are; fever, RUQ pain, jaundice, itching and portal hypertension, in patients who have grown up in rural areas with poor hygiene (7, 9-11).Early diagnosis is imperative in order to prevent complications (11). The basic imaging tools for the diagnosis of alveolar Echinococcosis are US and CT scans (12). US findings show large-sized, irregular cysts with heterogeneous content (12). We have had similar findings in all of our cases, although CT findings have also helped to characterize the number, size and calcification of the lesions (13).A final diagnosis is based on the histopathology findings, i.e., the presence of extensive necrosis and larval membranes, which are very characteristic (7). In two of our cases, the first biopsy was incorrectly diagnosed. In case No. 2, the larval membranes were incorrectly interpreted as hydatid cysts (Ecinococcus granulosus) and in case No. 3, the larval membranes were overlooked and the biopsy was reported as extensive necrosis, which was inadequate for an accurate diagnosis. Treatment of hepatic AE is extremely critical because, once the clinical symptoms of these patients appear; liver lesions have already become extensive making it hard to operate, so medical therapy is also very important (13). Surgical resection is the best treatment in the early stages of the disease, but only 40% of the cases can be operated with this organ-sparing intervention (11). Therefore, in this situation liver transplantation becomes the treatment of choice to cure the disease (14). In our three patients, the first one was inoperable, and albendazole treatment was started, and now after less than a year she is still alive and free of symptoms, unfortunately, she has not consented to have post-therapy imaging studies, in order to confirm a clinical improvement. Our second case was successfully treated with surgical resection, which is the best treatment, in operable cases (15).

In conclusion, these cases are rare reports of AE from Iran, which confirm the presence of this disease in the north of Iran and also to emphasize the presence of this disease in the southern regions as well. Our third case has never lived in the north, and it is the first report of AE from the Khuzestan Province in the south of the country. We would also like to emphasize the importance of the pathologic diagnosis of AE in the liver biopsies with extensive necrosis.
